# Cascaded neural network-based CT image processing for aortic root analysis

**DOI:** 10.1007/s11548-021-02554-3

**Published:** 2022-01-23

**Authors:** Nina Krüger, Alexander Meyer, Lennart Tautz, Markus Hüllebrand, Isaac Wamala, Marius Pullig, Markus Kofler, Jörg Kempfert, Simon Sündermann, Volkmar Falk, Anja Hennemuth

**Affiliations:** 1 Charité - Universitätsmedizin Berlin, Augustenburger Platz 1, 13353 Berlin, Germany; 2 German Heart Center Berlin, Berlin, Germany; 3 DZHK (German Centre for Cardiovascular Research), Partner Site Berlin, Berlin, Germany; 4 Fraunhofer MEVIS, Bremen, Germany; 5 Department of Health Science and Technology, Swiss Federal Institute of Technology, Zurich, Switzerland; 6 Darmstadt University of Applied Sciences, Darmstadt, Germany

**Keywords:** Deep learning, Image analysis, TAVI, U-Net, CNN, CT

## Abstract

**Purpose:**

Careful assessment of the aortic root is paramount to select an appropriate prosthesis for transcatheter aortic valve implantation (TAVI). Relevant information about the aortic root anatomy, such as the aortic annulus diameter, can be extracted from pre-interventional CT. In this work, we investigate a neural network-based approach for segmenting the aortic root as a basis for obtaining these parameters.

**Methods:**

To support valve prosthesis selection, geometric measures of the aortic root are extracted from the patient’s CT scan using a cascade of convolutional neural networks (CNNs). First, the image is reduced to the aortic root, valve, and left ventricular outflow tract (LVOT); within that subimage, the aortic valve and ascending aorta are segmented; and finally, the region around the aortic annulus. From the segmented annulus region, we infer the annulus orientation using principal component analysis (PCA). The area-derived diameter of the annulus is approximated based on the segmentation of the aortic root and LVOT and the plane orientation resulting from the PCA.

**Results:**

The cascade of CNNs was trained using 90 expert-annotated contrast-enhanced CT scans routinely acquired for TAVI planning. Segmentation of the aorta and valve within the region of interest achieved an F1 score of 0.94 on the test set of 36 patients. The area-derived diameter within the annulus region was determined with a mean error below 2 mm between the automatic measurement and the diameter derived from annotations. The calculated diameters and resulting errors are comparable to published results of alternative approaches.

**Conclusions:**

The cascaded neural network approach enabled the assessment of the aortic root with a relatively small training set. The processing time amounts to 30 s per patient, facilitating time-efficient, reproducible measurements. An extended training data set, including different levels of calcification or special cases (e.g., pre-implanted valves), could further improve this method’s applicability and robustness.

## Introduction

Transcatheter aortic valve implantation (TAVI) allows minimally invasive replacement of pathological aortic valves. However, purely image-based prosthetic valve selection is challenging.

**Fig. 1:**
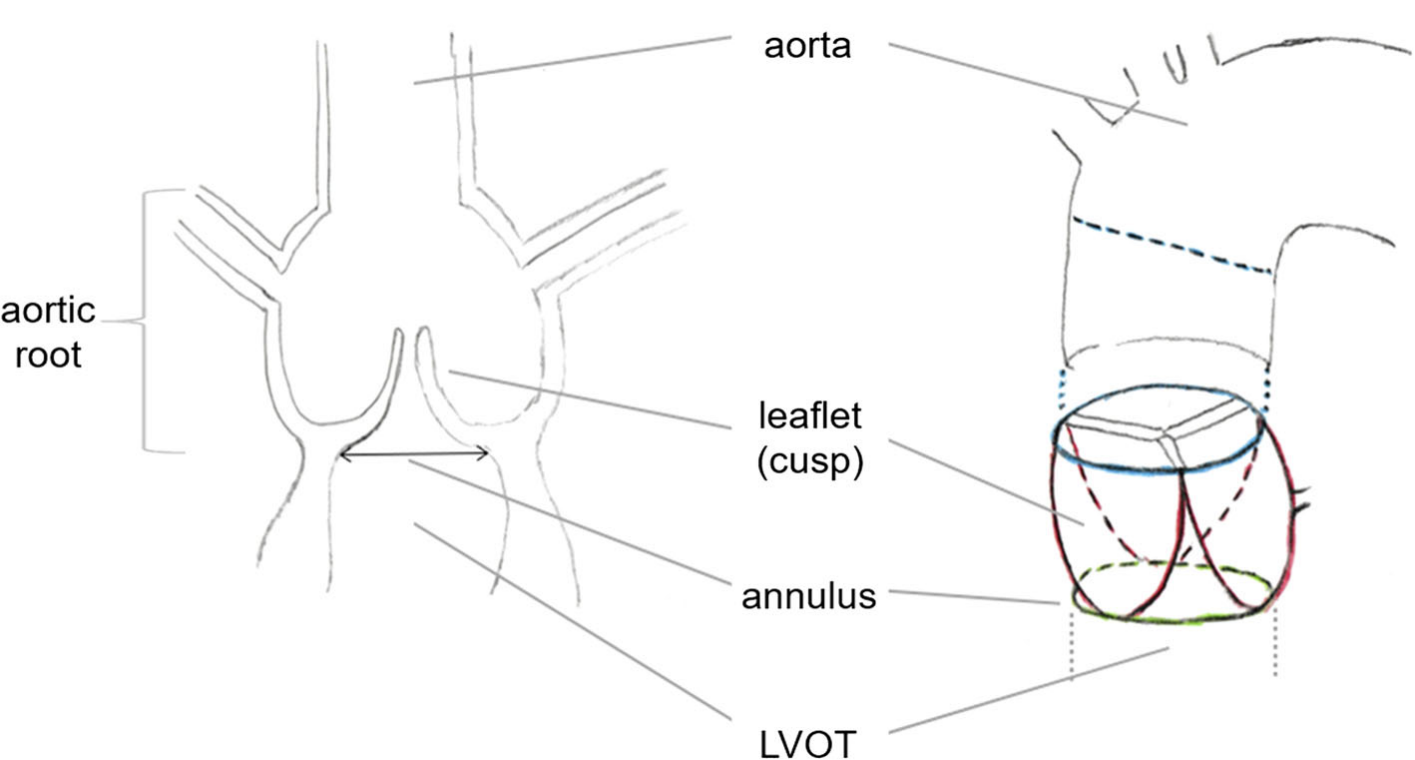
The aortic root comprises the whole aortic valve between the transition to the ascending aorta and the annulus at the base of the aortic valve cusps, which are attached to the LVOT

Figure 1 shows the complex anatomy of the aortic valve region. The implant needs to fit tight to the aortic wall and must not cover the coronary artery branches. Its orientation should be similar to the original valve. Therefore, it is essential to carefully analyze the aortic root before surgery to determine the best prosthesis and appropriate placement strategy. The assessment of the aortic valve annulus is crucial for prosthesis selection. Corresponding parameters such as the annulus diameter are highly sensitive to the orientation of the measurement plane. Several studies investigated the reliability and repeatability of interactive measurements: Meyer et al. [10] observed a difference in resulting prosthesis size between two software solutions in 18% of the considered patients. Schuhbaeck et al. [14] report mean inter-observer differences of $0.4\pm 0.9$ mm with a maximum of 5.6 mm for the area-derived annulus diameter.

**Fig. 2:**
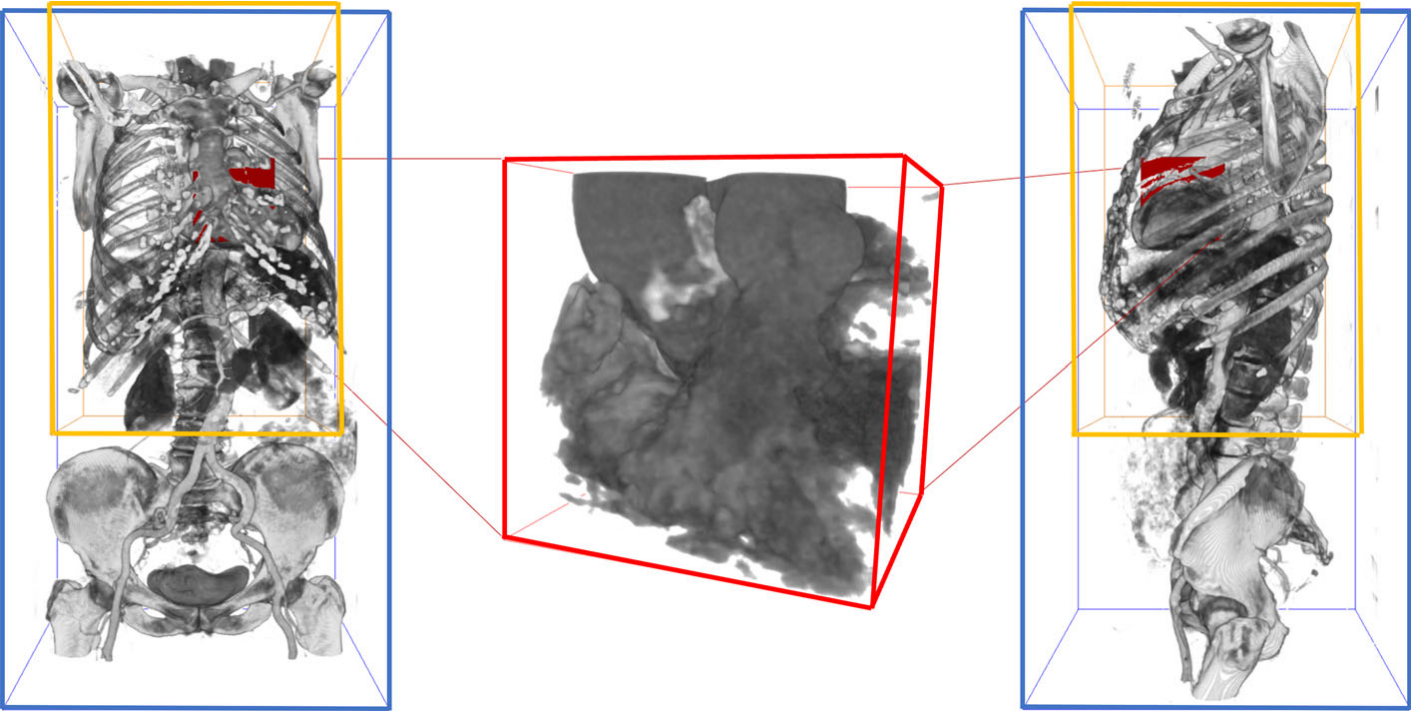
The blue bounding box contains the complete TAVI planning CT scan. The preprocessing for the aortic root detection crops this volume to the yellow region of $256\times 256\times 384$ mm$^3$ centered at a point 128 mm above the image center (in HEAD direction, according to the DICOM information). The subimage in the red bounding box contains the aortic root. The first task in this work is the automatic extraction of this subimage

**Fig. 3:**
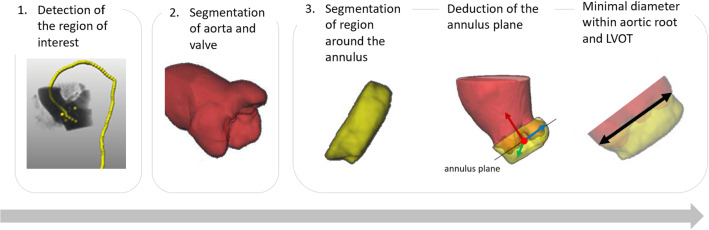
Processing steps: 1. The region of interest (ROI) is extracted from the thorax CT scan. 2. The aorta is segmented in this ROI. 3. The annulus region is segmented. The annulus plane is inferred by principal component analysis, and the annulus diameter is approximated

We aim to support aortic root analysis with a fully automatic, deterministic and patient-specific approach, facilitating standardization and reduction of effort for TAVI planning.

We break down the task into a cascade of neural networks as suggested for related contexts [12]:

1. Detection of a uniformly sized bounding box around the aortic valve;
2. Segmentation of the aorta including aortic valve within this bounding box;
3. Approximation of the area-derived annulus diameter based on the segmentation of the aortic valve and the annulus region.

We aim at developing an approach, applicable to a wide range of CT scanner models. We test our approach on expert-annotated routine clinical data acquired by different CT scanners and compare the suggested implant size based on the approximated annulus diameter with the implanted device size.

### Related work

This section discusses the reviewed literature on segmentation of the aortic root region in contrast-enhanced CT images. Lalys et al. presented a comprehensive pipeline for TAVI analysis, using atlas registration and deformable 3D snakes [8]. Their approach requires the placement of a point in the aortic root region. Elattar et al. used fuzzy classification and normalized cuts to segment the aortic root after detecting the centerline [3]. They reported smooth aortic root surfaces, possibly affecting accuracy in subsequent measurements. Gao et al. proposed atlas-based segmentation followed by surface model fitting, with atlas images from a publicly available database [4]. Waechter et al. segmented the aortic root region with a mean-shape model [15]. Astudillo et al. described a CNN-based segmentation in manually defined annulus planes [2].

The approaches from Astudillo et al. and Lalys et al. require manual interaction, hindering implementation as a fully automatic and deterministic processing tool. The aortic root models of Elattar et al., Gao et al. and Waechter et al. are smoothed or based on a smooth model. The model’s deformation is limited and thus only yields an approximation of the patient-specific anatomy.

Given the reliable performance of CNN methods in recent years, our aim is a conceptually simple and fully automatic pipeline consisting of multiple hierarchical CNN steps.

## Methods

We propose a neural network-based three-step segmentation approach for the anatomical structures of interest from CT images. The first step extracts the region of interest (ROI) around the aortic root and LVOT (see Fig. 2).

Each resulting subimage is resampled to an isotropic resolution of 0.6 mm. This is close to the original image resolution, which varies between 0.38 and 0.7 mm and ensures that processing with the given hardware is possible (see “Appendix”—hardware). In the second step, the aorta, including the aortic valve, is segmented within this ROI. This segmentation serves as additional input for estimating the annulus diameter. In the third step, the region around the aortic annulus is segmented, and the orientation of the annulus plane is determined using principal component analysis (PCA). Based on this information, we approximate the annulus diameter. Figure 3 visualizes the sequence of steps.

**Table 1:** Statistics: patient sex (F female, M male, U unknown); age, BMI and aortic valve calcification (mean ± standard deviation) for training, test and device sizing data set

	Training (90 cases)	Test (36 cases)	Sizing (640 cases)
Sex	60 F, 30 M	12 F, 18 M, 6 U	317 F, 323 M
Mean age	80.4 ± 10.2	80.8 ± 5.4	78.9 ± 9.4
Mean BMI [kg/m$^2$]	27.8 ± 6.2	25.5 ± 5.1	28.1 ± 15.7
AV calcification [mm$^3$]	240 ± 212	165 ± 288	205 ± 203

The volume of calcification was assessed within the aortic valve by thresholding the CT scan at a Hounsfield unit of 850

### Data

Our data set comprises annotated 3D thorax CT scans of 126 patients from the German Heart Center Berlin and the Charité TAVI registry. We used 36 cases for testing. The remaining 90 cases were repeatedly split into 75 for training and 15 for validation in sixfold cross-validation.

Further, we collected a data set of 640 patients, who successfully underwent a TAVI procedure, to test the suitability of our model for device selection. A procedure was deemed successful when no complications were documented. Here, we compared the size of the implanted valve with our approximated annulus diameter. Patient statistics can be seen in Table 1.

All CTs of the training and sizing data set were acquired with Siemens scanners. In the test set, nine cases each were acquired with Siemens, Toshiba, Philips and GE scanners.

**Fig. 4:**
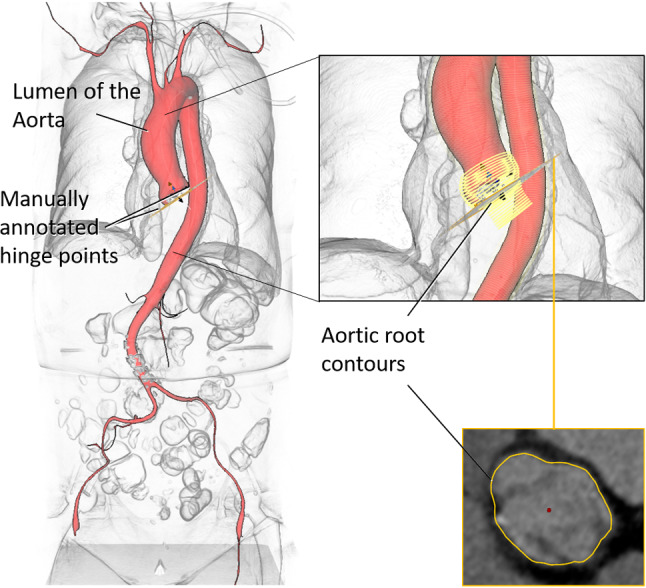
The available expert annotations consist of a mask of the aortic lumen, the centerline of aorta and LVOT, cross-sectional contours of the aortic root and LVOT and manually placed hinge point markers

The image volumes have slice dimensions of 512 $\times$ 512. The number of slices ranges from 273 to 1908, slice thickness 0.5–3 mm and spacing between slices 0.4–3 mm. The annotations shown in Fig. 4 were generated by domain experts using a custom MeVisLab^1^-based software prototype to obtain:

- a mask of the aortic lumen,
- the centerline through the aorta and LVOT,
- cross-sectional contours of the aorta and LVOT perpendicular to the centerline and
- three markers, indicating the hinge points of the aortic valve cusps.

The aorta mask includes lumen and valve without LVOT, and the cross-sectional contours show the outer contour of the aortic root and LVOT.

### Model training

**Fig. 5:**
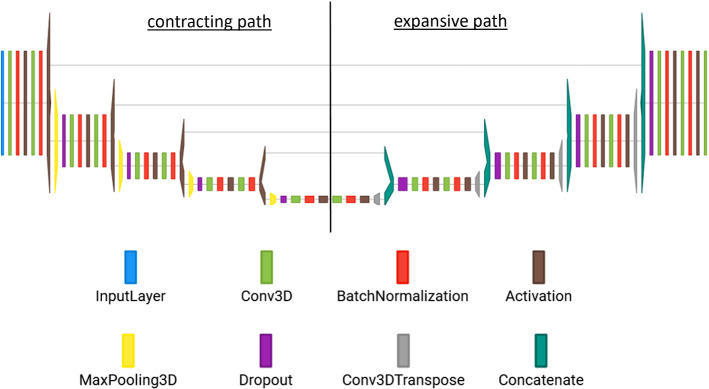
Our CNN architecture inspired by the U-Net: It consists of a contracting and an expansive path

For image processing, we considered the U-Net architecture, which has been successfully applied in several biomedical image segmentation challenges.^2^ We trained a structurally identical CNN for each processing step [11]. The specific layer arrangement is illustrated in Fig. 5.

It consists of a contracting path and an expansive path. In our application, the contracting path has nine convolutional layers combined with batch normalization, ReLU activation, max pooling and dropout. The expansive path alternates convolutions with transposed, also called up-convolutions. As the last layer, a $1\times 1$ convolution with sigmoid activation is used, resulting in a voxel-wise classification of the input image.

The neural network’s memory requirement depends on the number of filters, batch size and size of the input image. It has been shown that smaller batch sizes can improve accuracy and generalizability [5]. Thus, we selected a batch size of two for training. Given the size of the input images, the available hardware (see “Appendix”) allowed for a number of 18 filters in the first layer.

**Fig. 6:**
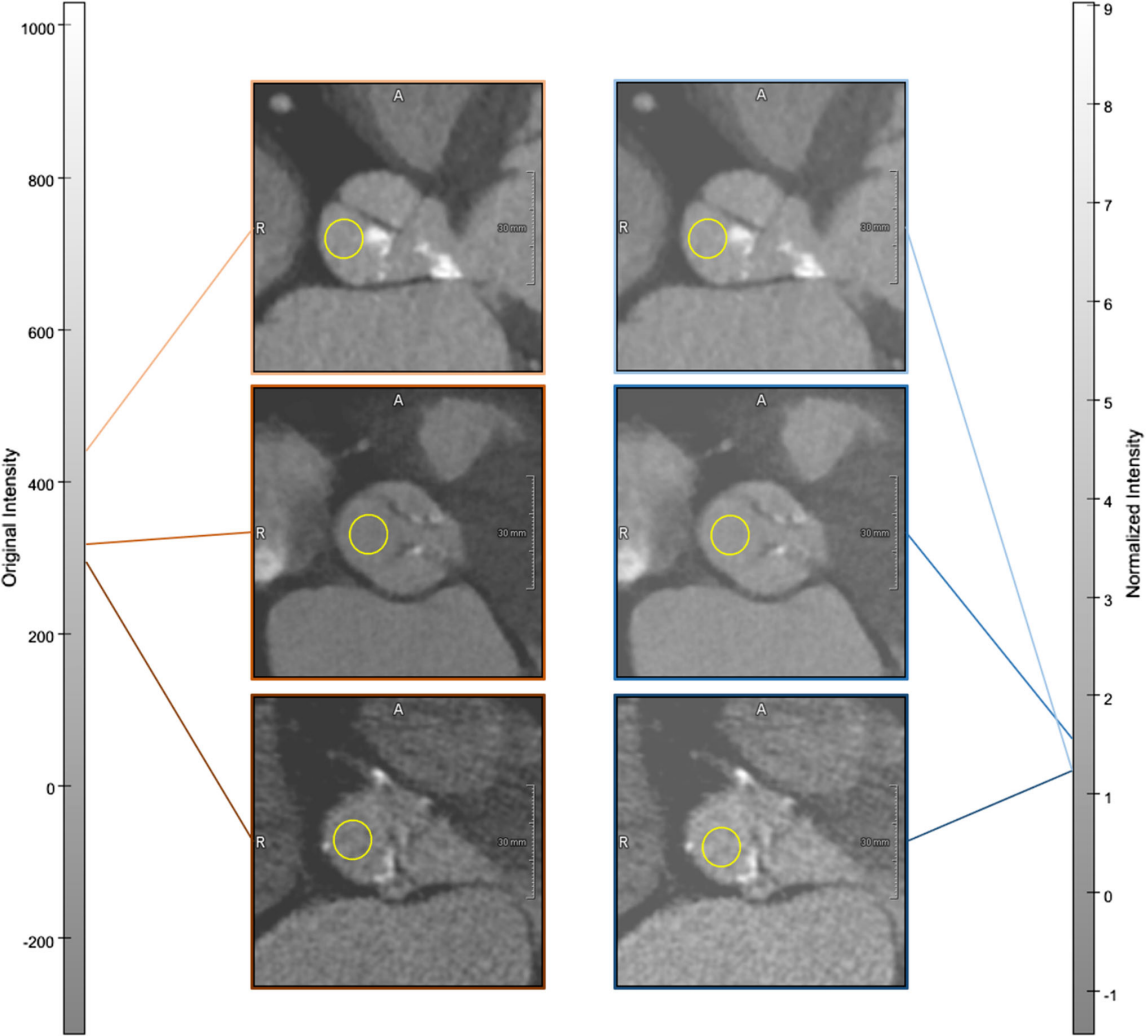
Example images acquired with different scanners. The relative intensity difference in the ROI (yellow circle) is reduced through the suggested intensity normalization. The lines mark the mean value within the ROI

The Hounsfield scale generally provides comparable intensities based on the tissue density. The usage of contrast agent, as well as differences in dosage settings, can, however, result in intensity variations. We apply intensity normalization using mean and standard deviation (see Fig. 6, for example).

In each epoch, each training image is augmented by translation. The image is randomly shifted between zero to five voxels in each direction. Because the patient orientation in the scanner is standardized, we performed no rotational augmentation. Deformations were not applied to avoid implausible anatomies.

The Adam optimizer [6] is used with different loss functions to find the best approach for each step. We considered the *binary cross-entropy*, as well as its variation, the *Focal Loss* [9], as we assume that the enhanced focus on the misclassified examples is appropriate in our scenario with the strong class imbalance.

- Binary Cross-Entropy
  $$\begin{aligned} L_{bc}(y, {\hat{y}}) = {\left\{ \begin{array}{ll} -log({\hat{y}}), &{} \text {if } y = 1\\ -log(1-{\hat{y}}), &{} \text {otherwise}, \end{array}\right. } \end{aligned}$$
- Focal Loss with $\gamma = 2$
  $$\begin{aligned} L_{f}(y, {\hat{y}})&= -(1-p_t)^{\gamma }log(p_t), \text {with} \\ p_t&= {\left\{ \begin{array}{ll} {\hat{y}}, &{} \text {if } y = 1\\ 1-{\hat{y}}, &{} \text {otherwise}, \end{array}\right. } \end{aligned}$$

where *y* is the target mask (1 if the voxel belongs to the segmentation and 0 otherwise) and ${\hat{y}}$ the predicted probability for each of the *n* voxels to belong to the segmentation.

We also evaluated the *Tversky Loss*, which is similar to the *F1 score*, but enables steering the emphasis on false-positive and false-negative classifications via the parameter $\beta$ [13], as well as the *Focal Tversky Loss* [1].

- Tversky Loss with $\beta \in [0.45, 0.50, 0.55,..., 0.95]$
  $$\begin{aligned} L_{t}(y, {\hat{y}})&= 1-T(y, {\hat{y}}), \text {with} \\ T(y, {\hat{y}})&= \frac{y{\hat{y}}}{y{\hat{y}} + \beta (1-y){\hat{y}} + (1-\beta )y(1-{\hat{y}})} \end{aligned}$$
- Focal Tversky Loss with $\beta \in [0.45, 0.50, 0.55,..., 0.95]$ and $\gamma = 2$
  $$\begin{aligned} L_{ft}(y, {\hat{y}}) = (1-T(y, {\hat{y}}))^{1/\gamma } \end{aligned}$$

An individual model for each step is obtained by selecting the four loss function and parameter combinations with the best results on a validation set of 15 patients after training on 75 patients, then training six structurally identical models in sixfold cross-validation per each of the four selected loss functions and averaging their outputs. We use this bagging approach to exploit the benefits of ensemble methods, exemplified by the results for step 1.

### Step 1: Aortic valve detection

In the first step, the image is cropped to a ROI around the aortic root and LVOT: The ROI detection is performed on a low-resolution (isotropic voxel size of 2 mm) subimage with an extent of $256\times 256\times 384$ mm$^3$ placed 128 mm above the image center (in the HEAD direction provided by the DICOM information) to enable fast memory-efficient processing. This image is padded if the upper border is outside the original image (see Fig. 2).

*Training * Based on the expert-defined aortic valve hinge points, an axis-aligned bounding box of $80\times 80\times 80$ mm$^3$ is placed in the image around the aortic root and LVOT, centered on the midpoint of the hinge points. This bounding box is used as a mask to learn the detection of the ROI.

**Fig. 7:**
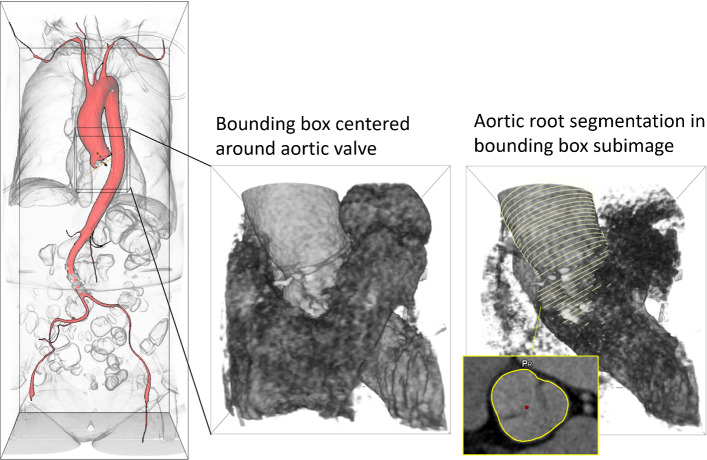
The labels for the training of the ROI segmentation are generated based on the aortic valve hinge points via the calculation of an axis-aligned enclosing bounding box. The labels for the ensuing aorta segmentation in step 2 are based on the aortic lumen, while the region around the annulus plane is defined by the cross-sectional contours of the aortic root and LVOT

*Segmentation postprocessing* For each voxel in the input CT, the CNN returns a probability for this voxel to belong to the uniformly sized ROI. This is binarized with a threshold of 0.5, and a bounding box is drawn around all predicted voxels. This box is resized to the desired dimensions of $80\times 80\times 80$ mm$^3$ with the midpoint being the center of the predicted bounding box.

### Step 2: Aorta segmentation

In the second step, a CNN is trained on the aorta segmentation within the ROI. The original images are resampled to an isotropic voxel size of 0.6 mm before being cropped to the ROI.

*Training* The label generation for this step is based on the aortic lumen within the ROI (see Fig. 7).

*Segmentation postprocessing* No postprocessing is required in this step.

### Step 3: Aortic root analysis

In the third step, the region around the aortic annulus plane, defined by the three hinge points, is segmented to deduce the orientation and midpoint of the annulus plane and enable measurements of the annulus region. As an output of step 3, the aorta segmentation from step 2 is reduced to the valve cusps and combined with the segmentation of the annulus region from step 3 to achieve a segmentation comprising valve cusps, annulus and LVOT. This segmentation is analyzed in cross sections oriented parallel to the derived annulus plane, and the diameter over all cross sections is measured to determine an approximation of the annulus diameter.

**Fig. 8:**
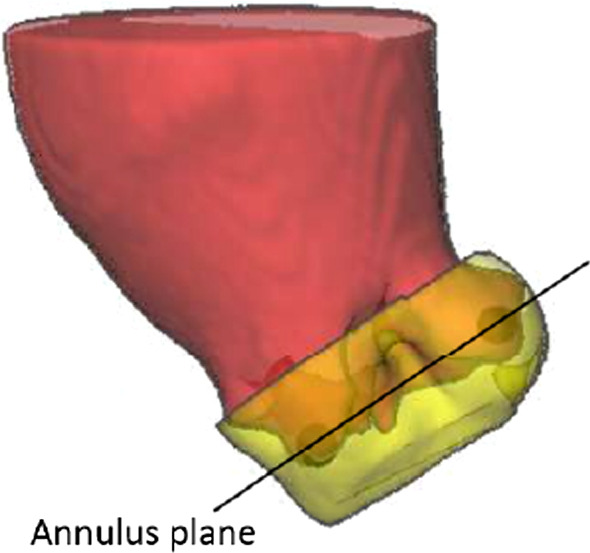
Segmentation mask (yellow), aligned along the annulus plane, defined by the hinge points (black) with the aorta segmentation (red) for reference

*Training* Based on the expert-defined hinge point markers (Fig. 4), we estimate the orientation of the annulus plane. To train the CNN, we consider the mask of the aortic root and LVOT along a 10 mm margin in the direction of the plane normal, as visualized in Fig. 8. The margin of 10 mm was selected experimentally to achieve a mask that can be successfully trained while also yielding an ideal approximation of the annulus plane from PCA. With this 3D approach, we intend to make our analysis robust against the input uncertainty of the hinge points.

**Fig. 9:**
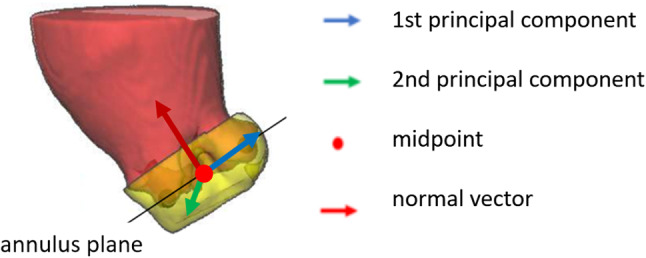
Principal component analysis applied to the segmentation mask (yellow): The first two principal components maximize the variance of the underlying data while being perpendicular to each other. A plane results from the normal vector orthogonal to the two principal components. The plane’s midpoint is defined by the center of gravity of the underlying data

*Segmentation postprocessing* A PCA is applied to deduce the annulus plane from the predicted segmentation (see Fig. 9). PCA is a dimensionality reduction method, which reduces a data set to its uncorrelated components that maximize variance. By definition of our annulus plane region, its diameter parallel to the annulus plane should be larger than its height. According to the literature, an annulus diameter typically exceeds 17 mm [14] and the height of our segmentation mask is defined to be 10 mm. Thus, the two components with the highest variance describe the orientation of the annulus plane. The vector orthogonal to the two largest eigenvectors of the PCA is taken as an estimate of the plane’s normal vector. The center of gravity of the predicted segmentation estimates the midpoint of the annulus plane. This midpoint and normal vector allow for approximation of the annulus plane. In our training data set, the mean Euclidean distance between the midpoint of the three hinge points and the midpoint deduced from PCA is 1.5 mm and the mean angle difference between the resulting normal vectors is $1.6^{\circ }$.

**Fig. 10:**
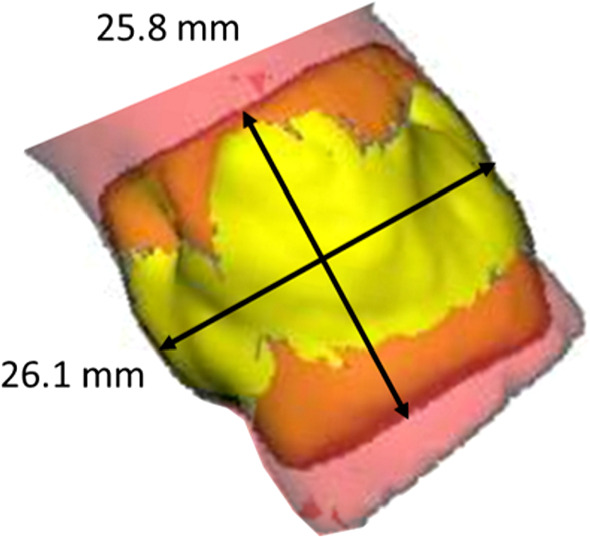
Calculation of the annulus plane orientation by principal component analysis can be distorted by a segmentation that has a comparable height and diameter; the minimal and maximal diameter should exceed the height to ensure correct calculation of the plane’s normal vector

However, the result is misleading if a segmented annulus region has a height comparable to its diameter (Fig. 10).

**Fig. 11:**
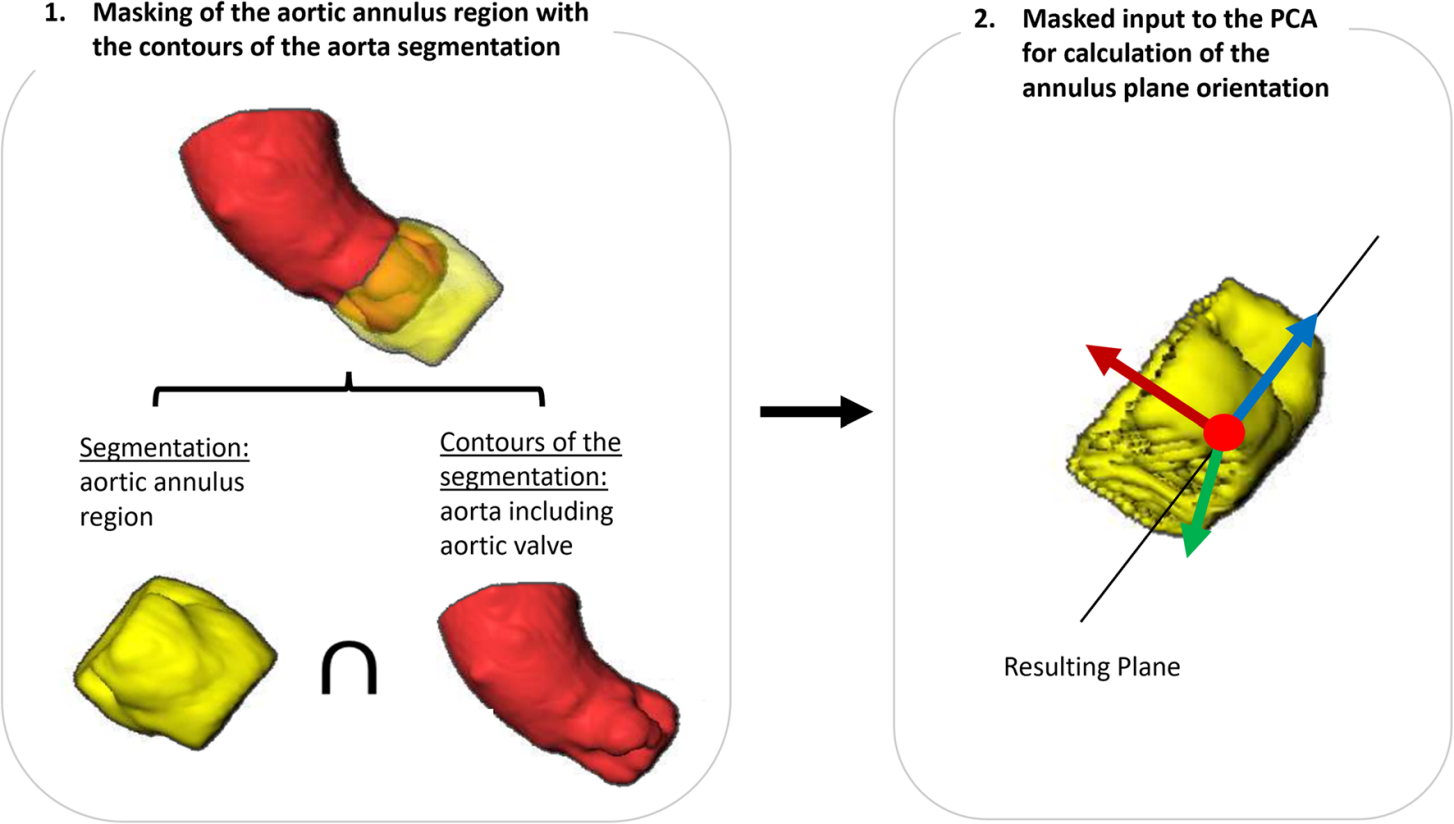
1. The segmentation of the annulus region is masked with the contours of the predicted aorta segmentation from step 2. 2. The resulting segmentation is used as input to the PCA for the calculation of the valve plane orientation

To ensure that the PCA detects the correct orientation, the predicted segmentation of the annulus region is masked with the contour of the segmentation from step 2 (see Fig. 11). Undesired segments are removed by only considering the largest connected component. The input of the PCA is thereby more accurately aligned along the annulus plane; however, the center of gravity is shifted into the valve. In our training data set, the mean Euclidean distance between the midpoint of the three hinge points and the midpoint deduced from this adjustment to the PCA input is 6.8 mm and the mean angle difference between the resulting normal vectors is $1.7^\circ$. The midpoint and normal vector resulting from the masked segmentation of the annulus region allow for the approximation of the annulus plane.

*Aortic Root Analysis* The aortic valve segmentation from the previous step is combined with the segmentation of the annulus region and reduced to a height of 10 mm along the resulting plane to approximate the aortic root region including aortic valve leaflets and LVOT. The minimal diameter within this region is calculated using the slice-wise convex hull contours aligned along the deduced annulus plane and obtaining the area-derived diameter for each. We only consider minima above 17 mm to ensure that all three aortic leaflets are contained within the contour; an aortic annulus diameter below 17 mm is unlikely [14]. The reference diameter, used as ground truth, is obtained in the same manner from the annotated data: The aortic valve and LVOT segmentation masks are intersected parallel to the plane defined by the hinge points, and the reference diameter is calculated as minimum area-derived diameter in the resulting cross sections within a range of $\pm 5$ mm along the plane normal.

In addition to the annotated data set, we apply our method to 640 CT scans of patients who underwent a successful TAVI. We compare the size of the implanted device to our suggestion based on our area-derived diameter, inspired by the sizing strategy from Schuhbaeck et al. [14]: a 23-mm valve for annulus diameters of 19.5–22.5 mm, a 26-mm valve for 22.5–26.5 mm, a 29-mm valve for 26.5–29.5 mm; we further suggested a 20-mm valve for annulus diameters below 19.5 mm and a 34-mm valve for greater than 29.5 mm.

## Results

The following subsections show the results per step.

### Step 1: Detection of the region of interest

In the detection of the ROI, a bounding box with a fixed size around the aortic root and LVOT centered around the hinge points shall be found by segmentation. The detected bounding box $BB_\mathrm{unet}$ is compared to the reference bounding box $BB_\mathrm{ref}$ using the intersection over ground truth (IoG):

$$\begin{aligned} \mathrm{IoG} = \frac{\mathrm{Intersection}}{\mathrm{Ground Truth}} = \frac{\left| BB_\mathrm{unet} \cap BB_\mathrm{ref}\right| }{\left| BB_\mathrm{ref}\right| } \end{aligned}$$

The resulting bounding box around the aortic root and LVOT is used as input for the following steps. The IoG is not directly applied to the predicted segmentation but to the bounding box obtained after thresholding and reshaping, as explained in the “Methods” section.

**Table 2:** Step 1: Mean intersection over ground truth for one model trained with each loss function, the selected four highlighted in italics

Model ($\beta$)	Training (75 patients)	Validation (15 patients)
Binary cross-entropy	0.963	0.923
Focal	0.931	0.912
Tversky (0.75)	0.970	0.929
*Tversky (0.80)*	*0.944*	*0.933*
Tversky (0.85)	0.973	0.909
Focal Tversky (0.60)	0.954	0.925
*Focal Tversky (0.65)*	*0.961*	*0.931*
Focal Tversky (0.70)	0.961	0.918
Focal Tversky (0.80)	0.969	0.927
*Focal Tversky (0.85)*	*0.952*	*0.932*
*Focal Tversky (0.90)*	*0.948*	*0.933*
Focal Tversky (0.95)	0.958	0.914

Table 2 shows the results for the individual loss functions reduced to the best parameters for clarity. For the final model, we selected the *Tversky Loss* with $\beta =0.80$ as well as the *Focal Tversky Loss* with $\beta =0.65$, $\beta =0.85$ and $\beta =0.90$, because they provide the highest IoG on the validation set (highlighted in italics). Table 3 shows the IoG for the selected loss functions evaluated on all 90 training samples and the 36 test samples.

**Table 3:** Step 1: Mean intersection over ground truth for each loss function in sixfold cross-validation and the final model as average of these models

Model ($\beta$)	Training (90 patients)	Test (36 patients)
Tversky (0.80)	0.951	0.936
Focal Tversky (0.65)	0.957	0.937
Focal Tversky (0.85)	0.954	0.938
Focal Tversky (0.90)	0.943	0.932
Final model	0.957	0.938

The final model resulting from the bagging of several models shows a higher IoG than any of the individual models in Table 2.

### Step 2: Aorta segmentation

In the second step, the aorta, including the aortic valve within the obtained bounding box, is segmented. The resulting mask $M_\mathrm{unet}$ is compared to the reference label $M_\mathrm{ref}$ with the F1 score (Dice coefficient), which considers precision $\frac{\left| M_\mathrm{unet} \cap M_\mathrm{ref} \right| }{\left| M_\mathrm{unet} \right| }$ and recall $\frac{\left| M_\mathrm{unet} \cap M_\mathrm{ref} \right| }{\left| M_\mathrm{ref} \right| }$:

$$\begin{aligned} F1 = 2 \cdot \frac{\mathrm{precision} \cdot \mathrm{recall}}{\mathrm{precision} + \mathrm{recall}} \end{aligned}$$

Table 4 shows the F1 score for the four loss functions reduced to the best parameters.

**Table 4:** Step 2: Mean F1 score with the individual loss functions, the selected four highlighted in italics

Model ($\beta$)	Training (75 patients)	Validation (15 patients)
Binary cross-entropy	0.943	0.935
Focal	0.922	0.920
Tversky (0.45)	0.925	0.913
*Tversky (0.50)*	*0.943*	*0.929*
Tversky (0.55)	0.933	0.926
Focal Tversky (0.50)	0.928	0.917
*Focal Tversky (0.55)*	*0.946*	*0.937*
Focal Tversky (0.60)	0.942	0.927
*Focal Tversky (0.65)*	*0.945*	*0.938*
*Focal Tversky (0.70)*	*0.946*	*0.938*
Focal Tversky (0.75)	0.938	0.932

Table 5 shows the F1 score obtained from the selected loss functions, *Tversky Loss* with $\beta =0.55$ and *Focal Tversky Loss* with $\beta =0.55$, $\beta =0.65$ and $\beta =0.70$, as well as the final model, which is again the averaged output of the models with these four loss functions.

**Table 5:** Step 2: Mean F1 score for each loss function in sixfold cross-validation and the final model

Model ($\beta$)	Training (90 patients)	Test (36 patients)
Tversky (0.50)	0.942	0.939
Focal Tversky (0.55)	0.945	0.936
Focal Tversky (0.65)	0.944	0.937
Focal Tversky (0.70)	0.944	0.940
Final model	0.945	0.940

### Step 3: Aortic root analysis

The aortic root is found by considering the segmented aortic valve and LVOT in cross sections reformatted to the orientation of the detected valve plane. The minimal area-derived diameter within the aortic root is compared to the minimal area-derived diameter obtained from the annotated data set. Table 6 shows the mean error, which was used for the selection of the optimal loss functions, the *Tversky Loss* with $\beta =0.55$, $\beta =0.85$ and $\beta =0.90$ and the *Focal Tversky Loss* with $\beta =0.80$.

**Table 6:** Step 3: Mean error in mm with the individual loss functions, the selected four highlighted in italics

Model ($\beta$)	Training (75 patients)	Validation (15 patients)
Binary cross-entropy	2.95	2.63
Focal	3.30	3.48
Tversky (0.50)	3.17	3.71
*Tversky (0.55)*	*1.96*	*2.49*
Tversky (0.60)	2.60	2.70
Tversky (0.80)	2.69	2.76
*Tversky (0.85)*	*2.15*	*2.6*
*Tversky (0.90)*	*2.06*	*2.52*
Tversky (0.95)	2.18	2.82
Focal Tversky (0.75)	2.96	3.01
*Focal Tversky (0.80)*	*2.59*	*2.62*
Focal Tversky (0.85)	2.01	2.64

Table 7 shows the results for the automatically obtained diameters from the final model, the diameters deduced from the annotations and the respective error. Table 8 compares the test results of the different CT scanner manufacturers. No significant difference between the errors for individual manufacturers can be observed in our test data set (ANOVA, $p =0.78$).

**Table 7:** Step 3: Mean minimal diameter ± standard deviation [minimal value, maximal value] (median)

	Training (90 patients)	Test (36 patients)
Model	$24.29 \pm 2.87$ [17.88, 31.99]	$24.11 \pm 2.57$ [19.29, 29.87]
From annotation	$22.84 \pm 3.61$ [17.86, 31.06]	$23.21 \pm 2.97$ [17.97, 31.25]
Error	$1.90 \pm 1.42$ [0.03, 5.10] (1.64)	$1.84 \pm 1.21$ [0.04, 4.00] (1.89)

All values are given in mm

**Table 8:** Step 3: Mean minimal diameter ± standard deviation in mm grouped by CT scanner manufacturer

	Siemens	Toshiba	GE	Philips
Error	$2.03 \pm 1.32$	$1.62 \pm 1.14$	$1.62 \pm 0.88$	$2.08 \pm 1.35$

**Table 9:** Step 3: Implant size suggestion versus actually implanted size in %

	Training (90 cases)	Test (36 cases)	Sizing (640 cases)
> 2 sizes smaller	1	0	3
2 sizes smaller	1	7	6
1 size smaller	14	25	17
*Correct size*	*81*	*61*	*64*
1 size bigger	1	6	5
2 sizes bigger	1	$<1$	3
$> 2$ sizes bigger	1	0	2

**Fig. 12:**
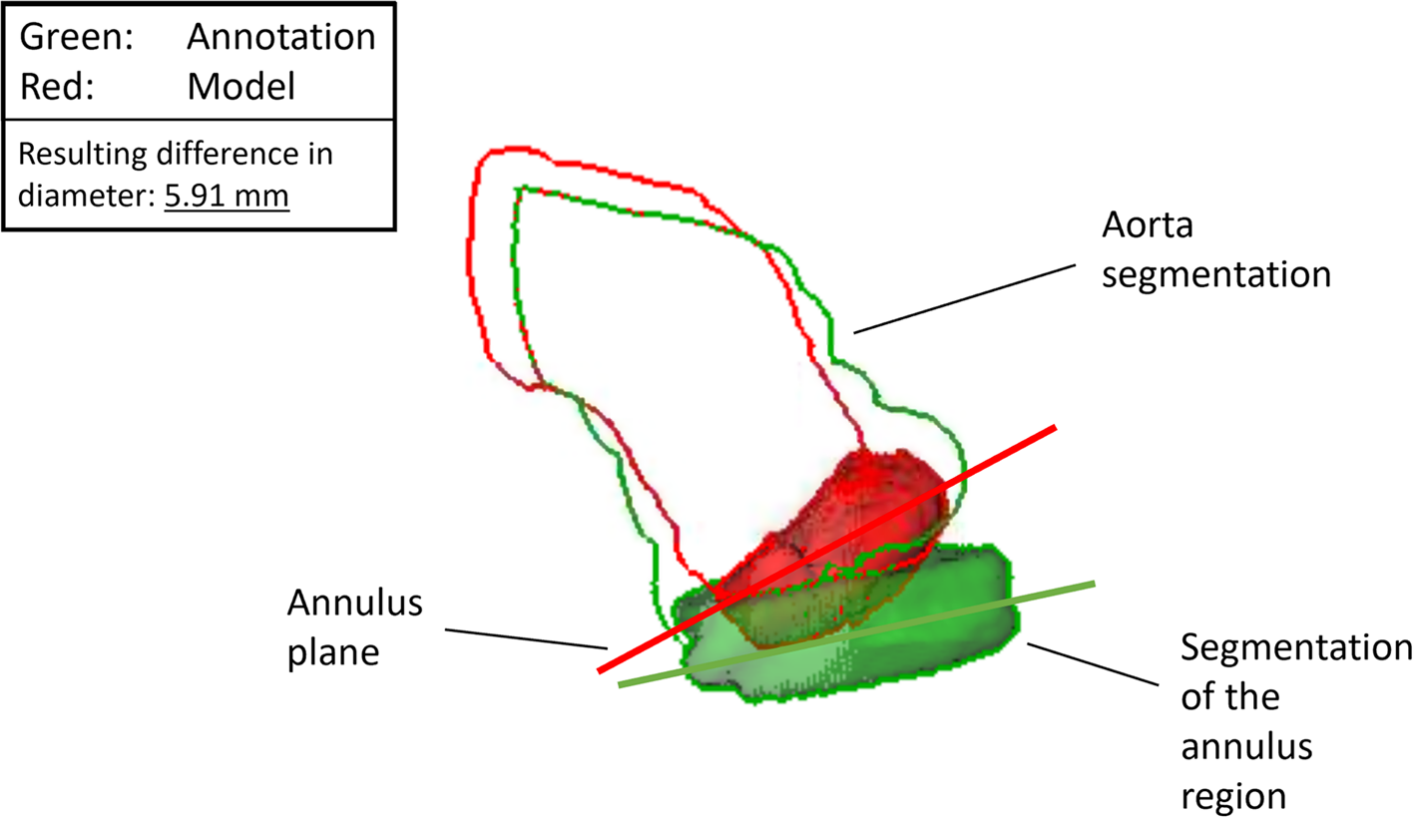
Possible error sources: a difference between the annotations (green) and the model (red) in detected plane orientation (lines), aorta and valve segmentation (silhouettes) and segmentation around the annulus plane (solid); in this example, this leads to a difference in the diameter obtained from the model and the annotations of 5.91 mm

Table 9 shows the results for implant size selection. We suggested the correct implant size in 81% of the training cases, 61% of the test cases and 64% of the device sizing cases.

On average, the processing time for one patient from the input of the original CT to the measurement of the minimal diameter amounts to 30 s, given the available hardware (see “Appendix”).

## Discussion and conclusion

This paper aims to test the suitability of a neural network-based approach to support aortic root analysis for TAVI planning. Cascaded neural networks are applied for three processing steps: detection of the ROI around the aortic root and LVOT, segmentation of the aorta, including the aortic valve, and segmentation of the region around the aortic annulus. The inferred minimal area-derived diameter within the annulus region is compared to the diameter from expert annotations. The aortic root is measured in cross sections, whose orientation corresponds to the annulus plane. The annulus plane is approximated by applying a PCA to the segmentation of the annulus region, masked with the outer edge of the aortic valve segmentation.

On average, our calculated minimal area-derived diameter within the annulus region deviates less than 2 mm from the minimal diameter from expert annotations. The calculated diameters are in a range comparable to similar studies [3, 8, 15]. The implant size could be inferred in 81% of the cases for the training set, 62% for the test set and 64% for the device sizing set. No significant difference between different CT scanners could be observed.

The accuracy of the model’s predictions depends on the annotations of the data set. For the present study, the training set comprises a relatively small number of 90 cases and 36 cases are used for the evaluation of the model. Further improvement and evaluation with a larger data set are advisable.

Future work is also required to optimize the detection of the aortic annulus plane. We currently only consider the plane orientation, while its exact position is estimated by the minimal area-derived diameter in the annulus region.

The measured diameters are highly dependent on several factors, such as the orientation of the detected annulus plane. Kütting et. al report that plane tilting leads to a different choice in valve prosthesis size in 33% of cases for $5^{\circ }$ of tilt and 66% of cases for $10^{\circ }$ [7]. Figure 12 shows an example of possible error sources—a difference in detected plane orientation, aortic valve segmentation and segmentation around the annulus plane. In this example, all error sources are present.

The optimal valve size does not only depend on the annulus diameter but also other factors, such as the coronary distances, calcifications or aortic dilation. Although the strategy for prosthesis sizing in the clinical setting currently also concentrates on the annulus measurement, we suggest consideration of the more complex three-dimensional aortic root anatomy by, for example, regarding several planes throughout the aortic root along the centerline. This would allow for a thorough analysis of the aortic root contour and diminish the effect of measurement of a single tilted annulus plane. For such an extensive aortic root analysis, an automatic approach would be highly valuable for time efficiency and reproducibility of results.

The current results suggest neural network-based aortic root analysis as a promising approach to support fully automatic, time-efficient and reproducible aortic root assessment for TAVI device sizing and selection, which can be improved through enhanced training data on the one hand and more sophisticated consideration of the aortic root anatomy on the other hand.

## Data Availability

Not publicly available.

## Appendix

### Hardware and software specifications

A Linux server with the following hardware specifications was used for model development:

- 2 Intel Xeon Gold 5215 @ 1.7 GHz,
- 4 nVidia Titan V 12 GB HBM2 (GV100, SM7.0).
- 96 GB RAM

Depending on the regarded step, the average training time of one CNN ranged between 20 minutes and one hour.

We used Keras 2.3.1 with the TensorFlow backend (tensorflow-gpu 2.1.0).

## Footnotes

1 https://www.mevislab.de/mevislab/.

2 The ISBI 2015: https://biomedicalimaging.org/2015/, where the U-Net won two challenges: https://lmb.informatik.uni-freiburg.de/people/ronneber/isbi2015/.

## Declarations

### Code availability

Not publicly available.
